# Enhanced N-Glycan Profiling of Therapeutic Monoclonal Antibodies through the Application of Upper-Hinge Middle-Up Level LC-HRMS Analysis

**DOI:** 10.3390/antib13030066

**Published:** 2024-08-06

**Authors:** Natalia Mesonzhnik, Anton Belushenko, Polina Novikova, Alexey Kukharenko, Mikhail Afonin

**Affiliations:** 1 Resource Centre of Analytical Methods, Laboratory Complex, Sirius University of Science and Technology, Olympic Ave. 1, 354340 Sochi, Russia; repin.polina@gmail.com (P.N.); afonin.mb@talantiuspeh.ru (M.A.); 2 Federal Hygienic and Epidemiological Center of Rospotrebnadzor, Varshavskoe Highway 19a, 117105 Moscow, Russia; belushenkoao@fcgie.ru; 3 Laboratory of Pharmacokinetics and Metabolomic Analysis, Institute of Translational Medicine and Biotechnology, I.M. Sechenov First Moscow Medical University, 8/2 Trubetskaya, 119991 Moscow, Russia

**Keywords:** mAb, LC-HRMS, middle-up, intact MS, enzymes, IdeS, gingipain K, IgdE, intact MS, N-glycosylation

## Abstract

Therapeutic monoclonal antibodies (mAbs) are crucial in modern medicine due to their effectiveness in treating various diseases. However, the structural complexity of mAbs, particularly their glycosylation patterns, presents challenges for quality control and biosimilarity assessment. This study explores the use of upper-hinge middle-up (UHMU)-level ultra-high-performance liquid chromatography–high-resolution mass spectrometry (LC-HRMS) analysis to improve N-glycan profiling of mAbs. Two specific enzymes, known as IgG degradation enzymes (IGDEs), were used to selectively cleave therapeutic mAbs above the hinge region to separate antibody subunits for further Fc glycan analysis by means of the UHMU/LC-HRMS workflow. The complexity of the mass spectra of IGDEs-digested mAbs was significantly reduced compared to the intact MS level, enabling reliable assignment and relative quantitation of paired Fc glycoforms. The results of the UHMU/LC-HRMS analysis of nine approved therapeutics highlight the significance of this approach for in-depth glycoform profiling.

## 1. Introduction

The development of biopharmaceuticals, particularly based on monoclonal antibodies (mAbs), is a significant and central area within the pharmaceutical industry [1,2,3,4,5]. This prominence is due to their clinical efficacy and targeted specificity in the treatment of socially important diseases, including but not limited to diabetes, cancer, cardiovascular diseases and autoimmune diseases [6,7,8,9,10,11].

The specificity of antibodies to relevant antigens and the ability to identify a wide range of biological targets has led to a rapid increase in the number of mAb drug candidates worldwide. As of 2022, more than 90 mAb products have been approved for use in Europe, and over 100 have been approved for use in the US [5,12,13]. These drugs are biotechnologically produced in predefined cell lines. During the production, purification or storage of mAbs, post-translational modifications (PTMs) or other structural changes occur, resulting in diverse forms of the final product. The combination of multiple proteoforms leads to macro- or microheterogeneity of antibodies. Variations in the composition of certain PTMs, such as oxidation, deamidation or glycosylation, can alter the biological activity of a drug and impact its stability and safety profiles [14,15,16,17,18].

Most therapeutic mAbs are derived from human immunoglobulin type 1 (IgG1) and are glycosylated in a conserved region of the crystallizing fragment (Fc) of the heavy chain (HC). The glycosylation of this region determines many critical properties of an antibody, such as its interaction with the immune system via Fc receptors and the complement system, pharmacokinetic profile and immunogenicity [15,16]. Therefore, licensing authorities in different countries have strict requirements to demonstrate glycan composition as part of quality control (QC) and for the approval of biosimilar versions of mAbs. Compliance with these requirements is ensured through the use of an arsenal of analytical methods [19].

Mass spectrometry (MS) is widely acknowledged as a primary tool for the characterization of protein molecules, including antibodies [20,21]. According to the recommendations of the ICH Q6B international harmonization conference, MS-based methods are mandatory for mAb characterization as part of the QC process [22]. 

MS-based analytical strategies involve analyzing mAbs at the intact level or with separation of the antibody into subunits (middle-up, middle-down) or smaller fragments (bottom-up) [23,24,25]. When analyzing antibody fragments, glycan structures are studied either as part of glycopeptides or larger fragments or separately as oligo- (released glycans) or monosaccharides. The bottom-up approach is traditionally used to identify glycosylation sites and to assess the heterogeneity of glycoforms at a specific site [25].

The characterization of glycosylation at higher levels is of particular interest for assessing the macroheterogeneity of the produced mAbs. This method provides information on the ‘average’ glycoprofile, i.e., the distribution of glycan species throughout the molecule as a whole or, more specifically, across its domains [25]. Intact MS analysis is one of the fastest and most available methods for assessing glycan heterogeneity using MS [24]. The analysis is typically conducted under denaturing conditions; nevertheless, its efficacy may be hindered by the simultaneous presence of various proteoforms within the mass spectra, particularly in multi-charge states, which cannot be effectively resolved due to technical constraints. Overlapping MS signals can deteriorate the signal-to-noise ratio, reducing the accuracy of deconvolution and, consequently, the correctness of glycoform annotation [19]. This method is commonly employed to analyze the high-level species (G0F/G0F, G0F/G1F, G1F/G1F, G1F/G2F and G2F/G2F), for example, as part of biosimilarity studies [19,23]. Native MS can increase the number of identified forms. However, this analysis is typically low-throughput, requiring large sample inputs and high-end mass spectrometers. 

Middle-up approaches are a favorable alternative for obtaining information on the modification of individual antibody subunits [26,27,28,29,30], particularly for mAbs with complex structures. The antigen-binding fragment (Fab) of cetuximab has additional glycosylation sites [31,32]. Antibody–drug conjugates (ADCs) are also used [33,34,35] along with bispecific antibodies [36] and Fc-fusion proteins [37,38,39,40].

**Figure 1:**
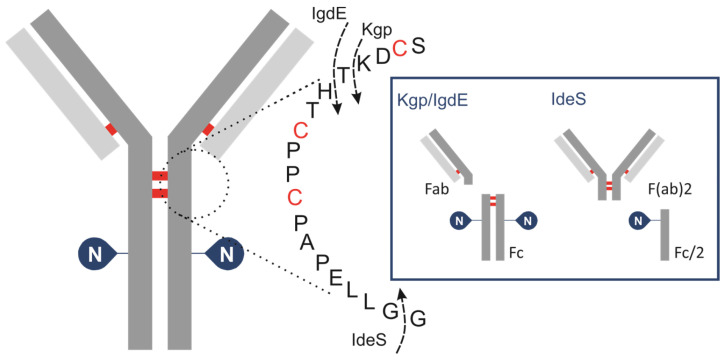
Schematic of human IgG, zooming in on hinge area, showing cleavage sites for IGDEs: IdeS, Kgp, IgdE and SpeB.

To achieve a controlled fragmentation process, subunits of IgG are produced by cleaving it with specific IgG degradation enzymes (IGDEs). Pepsin and papain are not recommended for middle-up analysis of mAbs due to their broad specificity, resulting in heterogeneous fragment generation and often incomplete digestion, which complicates result interpretation and may lead to artifacts such as aggregation. Enzymes with defined cleavage sites are preferred for this analytical approach to ensure controlled and reproducible fragmentation (Figure 1). 

IdeS protease, derived from Streptococcus pyogenes, is typically used to cleave IgG below the hinge region between the two glycine residues …CPPCPAPAPELLG/GPSVF… This results in the formation of an F(ab)′2 fragment (~100 kDa) and a glycosylated Fc/2 fragment (~25 kDa). Although this approach allows for the estimation of the oligosaccharide profile of the Fc/2 fragment, it results in the loss of important information regarding the pairing of glycoforms. A limited number of studies have investigated the application of IgG-degrading enzymes SpeB (Streptococcal pyrogenic exotoxin B) and Kgp (Lysine-specific gingipain/gingipain K). Sjögren et al. (2016) provide a detailed description of their properties [41]. This type of protease hydrolyses peptide bonds above the hinge region while retaining disulfide bonds that connect the HC of the IgG molecule. The resulting fragments from SpeB/KgP digestion are smaller and less complex compared to those analyzed by intact LC-MS but also retain the glycosylation information of both HCs of the antibody, unlike IdeS. The enzyme SpeB generates Fab and Fc fragments from mouse IgG and exhibits optimal activity under reducing conditions, and employing stronger reducing conditions increases the likelihood of reducing interchain thiols. Kgp operates under mild reducing reaction conditions, requiring only 2 mM cysteine, which is notably gentler compared to the conditions typically needed for SpeB. A new cysteine protease, IgdE, has recently been discovered [42]. It has the ability to cleave human IgG1 at a specific site above the hinge region without requiring reducing conditions. While it represents the first choice for processing human IgGs, its catalytic efficacy on Fc-fusion proteins is considerably diminished. The use of IgdE and SpeB for pharmaceutical antibody glycosylation characterization is poorly described in the literature. 

In this study, we compare the capabilities of two IgG-degrading proteases followed by LC-MS analysis to characterize nine approved therapeutic mAbs, with a focus on Fc glycan profiling at the upper-hinge middle-up level (UHMU LC-MS).

## 2. Materials and Methods

### 2.1. Materials, Chemicals and Reagents

LC-MS-grade acetonitrile (ACN) was obtained from PanReac AppliChem (Barcelona, Spain), 2-amino-2-(hydroxymethyl)-1,3-propanediol (≥99.9%, TRIS base) was purchased from Sigma Aldrich (Wicklow, Ireland) and formic acid (Optima LC/MS grade, ≥99.5%) (FA) and water (Optima LC-MS grade) were acquired from Fisher Chemical (Pittsburg, PA, USA). IdeS enzymes were obtained from Promega (Madison, WI, USA), and IgdE (FabALACTICA^®^) and Kgp (GingiKhan^®^) enzymes were both acquired from Genovis Inc. (Cambridge, MA, USA). Additionally, standard peptides, such as [Glu1]-fibrinopeptide B, mAb Mass Check Standard and mAb Subunit Standard, were obtained from Waters Corporation (Milford, MA, USA) and sodium iodide (99.999% trace metals basis) (SodY) was obtained from Sigma Aldrich (Wicklow, Ireland). Amicon Ultra-0.5 mL centrifugal filters were purchased from Merck (Darmstadt, Germany).

### 2.2. mAbs

Adalimumab (Humira^®^) was obtained as a solution for subcutaneous injection (50 mg/mL) from Wetter Pharma-Vertriebs GmbH & Co KG (Wiernsheim, Germany). Golimumab (Simponi^®^) was procured from Janssen Biotech, Inc. (Horsham, PA, USA) as a solution for subcutaneous injection at a concentration of 25 mg/mL. Trastuzumab (Herceptin^®^) was acquired as a lyophilized powder concentrate to be used in solution for infusion (21 mg/m) from F. Hoffmann-La Roche Ltd. (Basel, Switzerland). Rituximab (Mabthera^®^) was sourced as a concentrate to be used in solution for infusion from Roche Diagnostics GmbH (Basel, Switzerland), with concentrations of 10 mg/mL. Regdanvimab (Regkiron^®^) was obtained as a concentrate for infusion from Samsung BioLogics (Incheon, Republic of Korea), providing 60 mg/mL of the active compound. Tixagevimab and cilgavimab (Evusheldy^®^) were sourced from Samsung BioLogics/AstraZeneca AB (Incheon, Republic of Korea). The solution for intramuscular injection contained 100 mg/mL of each monoclonal antibody. Etesevimab and bamlanivimab were procured as solutions for infusion from Eli Lilly (Indianapolis, IN, USA), with a concentration of 35 mg/mL each. 

### 2.3. Antibody Digestion

All IGDEs were reconstituted in dH_2_O according to the manufacturer’s instructions: 50 units/µL IdeS, 40 units/μL IgdE and 10 units/μL Kgp. In case of Kgp, 10× reducing agent (cystein) was freshly prepared in dH_2_O at final concentration of 20 mM before each digestion. Sample aliquots containing 20 µg of protein were prepared in the appropriate digestion buffer solution at a substrate-to-enzyme ratio of 1 µg:1 unit. 

IdeS digestion was performed under nonreducing conditions in 25 mM Tris buffer (pH 7.5) or using Kgp in 100 mM Tris buffer (pH 8.0) with mild reducing conditions (2 mM cysteine) for 2 h at 37 °C. Enzymatic proteolysis with IgdE was performed in 25 mM Tris buffer (pH 7.5) overnight at 37 °C. After digestion, samples were buffer-exchanged using centrifugal filters Amicon Ultra 0.5 mL–30 kDa and diluted to 0.2 mg/mL with 0.1% formic acid in water prior to analysis. 

### 2.4. Instrumental Analysis

The characterization of intact mAbs and fragments generated by enzymatic digestion was performed using an I-Class ACQUITY UPLC system (Waters, Milford, MA, USA) coupled to an electrospray time-of-flight mass spectrometer (Xevo^®^ Q-ToF, Waters, Milford, MA, USA).

The mobile phase consisted of 0.1% FA (*v*/*v*) water solution (A) and 0.1% FA (*v*/*v*) in ACN solution (B). One µL (200 ng) of mAb sample was analyzed using a Waters ACQUITY UPLC BEH C4 column (ACQUITY UPLC Protein BEH C4, 300 Å, 1.7 µm, 2.1 mm × 50 mm). The column temperature was 80 °C, and the injection volume was 1 µL of mAb solution (0.2 µg of mAb). The mAbs were analyzed using a linear gradient of 15% to 55% B in 20 min (subunits) or 5% to 50% B in 3 min (intact MS) with a flow rate of 0.2 mL/min.

The mass spectrometer was operated in positive sensitivity mode and ions were scanned within an *m*/*z* window ranging from 500 to 4000 with a 1 s scan rate. Source/desolvation temperatures were maintained at 135 °C/500 °C for subunit analysis and 150 °C/600 °C for intact MS, respectively, and gas flow was set to 600 L/h. The capillary was set at 2.75 kV and the sampling cone at 100 V (subunits) or 140 V (intact MS).

The mass spectrometer was calibrated using the singly charged ions produced by NaI 2 µg/mL in a 50% 2-isopropanol/water solution. The Lockspray calibration was performed using the doubly charged ion (*m*/*z* 785.8421) produced by the 300 pmol/μL solution of [Glu1]-fibrinopeptide B in 50%/50%/0.1% water/ACN/FA. 

### 2.5. Data Analysis 

Data were processed and analyzed using the intact mass analysis workflow within the Biopharamceutical Platform Solution of UNIFI software v.1.8 (Waters, Milford, MA, USA). Average masses were calculated based on the protein sequences and expected modifications. For all mAbs, lysine clipping was set as C-terminal modification.

Chromatograms were generated based on total ion currents (TICs). The mass spectra for each mAb or mAb subunit from LC peaks were automatically deconvoluted using the MaxEnt1 algorithm and the resulting molecular weights (MWs) were matched to protein species during automated UNIFI data processing (using a 30 ppm mass accuracy threshold). The relative abundances of glycosylated species were determined using the integrated MS responses of the deconvoluted mass peaks.

## 3. Results

First, nine approved therapeutic mAb (Table S1) products were analyzed using LC-HRMS at the subunit level with two specific IgG degradation enzymes, IgdE and Kgp (Table 1, Figure 1). 

This technique has been used to selectively cleave the mAbs’ upper-hinge region, releasing dimeric Fc and Fab fragments of almost 50 kDa each. To corroborate the results of the UHMU approach, a middle-up analysis of mAbs using IdeS digestion was performed. This method involves cleavage of mAbs below the hinge region, yielding F(ab)′2 (approx. 110 kDa) and Fc/2 (approx. 25 kDa) fragments (Figure S1, Table S2).

### 3.1. IGDE Specificity Evaluation

**Figure 2:**
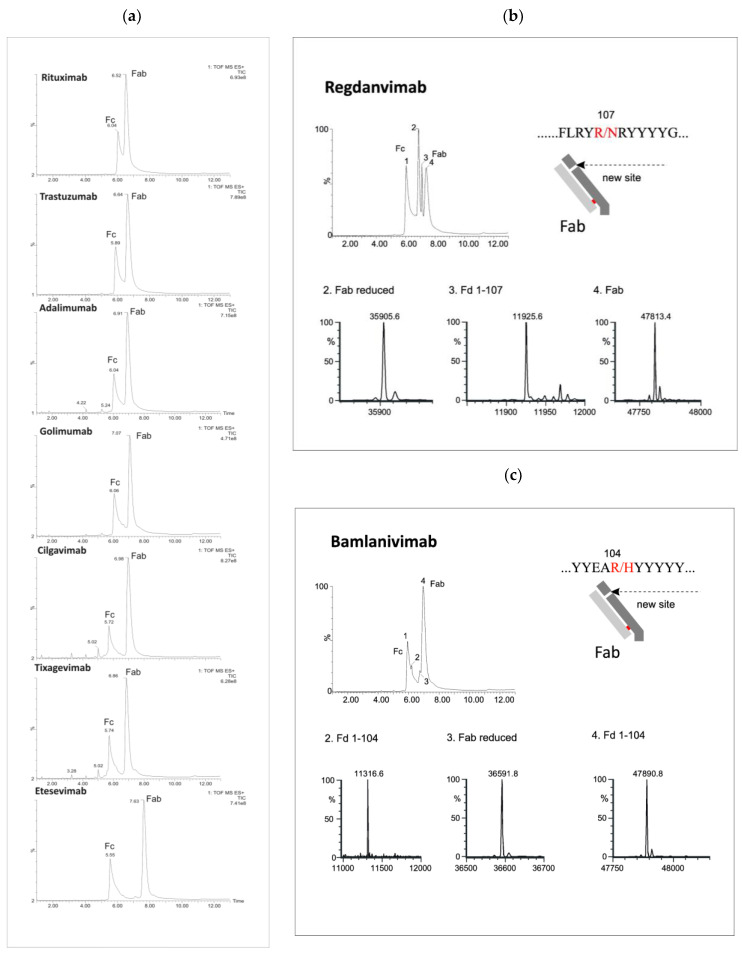
LC/MS profiles of the mAb fragments after digestion using Kgp (**a**) and identification of nonspecific cleavages of Fab fragments in regdanvimab (**b**) and bamlavinimab (**c**).

**Figure 3:**
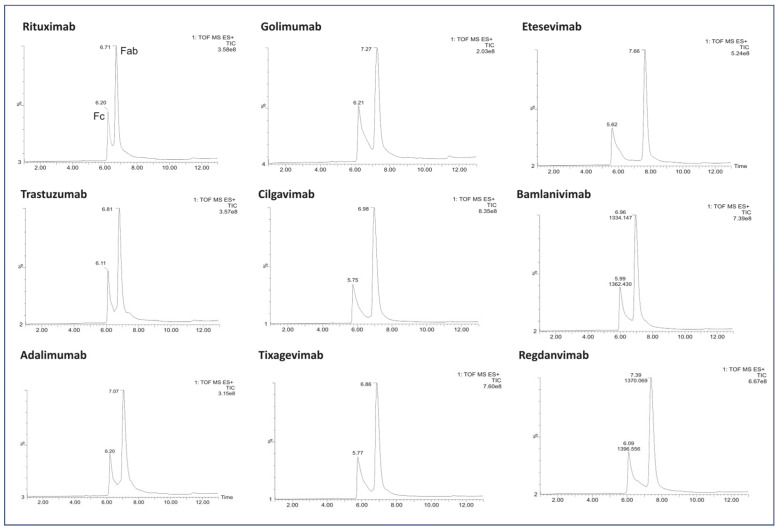
LC/MS profile of the mAb fragments after digestion using IgdE.

The LC/MS profiles obtained following enzymatic digestion revealed characteristic peaks corresponding to Fc and Fab fragments, indicating successful cleavage above the hinge region using both IgdE and Kgp enzymes (Figure 2 and Figure 3). 

Beyond the distinctive peaks corresponding to the Fc and Fab fragments, the chromatograms of Kgp-treated samples exhibited additional peaks, suggesting potential nonspecific cleavage reactions (Figure 2). Particularly, treatment of regdanvimab with the Kgp enzyme resulted in the formation of four different fragments. The results revealed the molecular weights (MWs) of peaks 1 and 4 to be 53,029 kDa and 478,134 kDa, respectively, corresponding to the values of the Fc and Fab subunits calculated from their amino acid (a.a.) sequences. Additionally, smaller fragments with MWs of 359,056 kDa and 119,256 kDa were detected in the Kgp digest (peaks 2 and 3). The sum of these fragments was equal to 478,312 kDa (MW of Fab plus a water molecule). This observation indicates that regdanvimab could undergo proteolytic cleavage by Kgp at Arg107. A similar phenomenon was observed with another human antibody, bamlanivimab. The degradation products of the Fab region were less abundant and presumably resulted from the breakdown of the Arg104-His105 amino acid bond of the bamlanivimab Fab chain. 

Gingipain K preferentially cleaves peptide bonds after lysine residues. The unspecific cleavage of these mAbs by Kgp can be associated with the primary and/or secondary structure of the antibodies’ CDR3 region. Regdanvimab (ARIPGFLRYRNRYYYYGMDV) and bamlanivimab (ARGYYEARHYYYYYAMDVW) both have multiple arginine residues in their extended CDR3 sequences (approx. 20 a.a.). Some of these residues are located near the middle of the region and may be more exposed and available for Kgp to cleave, as they are part of the antigen-binding site or the loop structure.

Arginine residues located further away from the midpoint of the loop may be less susceptible to protease interaction. For example, golimumab (DRGIAAGGNYYYYGMDV) has only one arginine residue in its 17-amino acid CDR3 sequence, which is located towards the end of the loop. This residue may not be easily accessible to Kgp. Therefore, the Fab cleavage products of golimumab were not observed by Kgp or were not significant enough to be detected. 

In addition, a number of low-intensity peaks were observed before the elution of the main peak of the Fc fragment. The molecular weights determined by LC-HRMS analysis identified these products as Fc degradants released by hydrolysis after lysin residuals at the CH2 and CH3 domains. For example, the most intense peptide fragment of tixagevimab and cilgavimab (RT = 5.02) belongs to the cleavage between the last CH2 lysine (Figure S2, Table S3). 

Importantly, the absence of additional chromatographic peaks was conspicuous following proteolysis by IgdE (Figure 3).

### 3.2. N-Glycoprofiling of mABs

**Figure 4:**
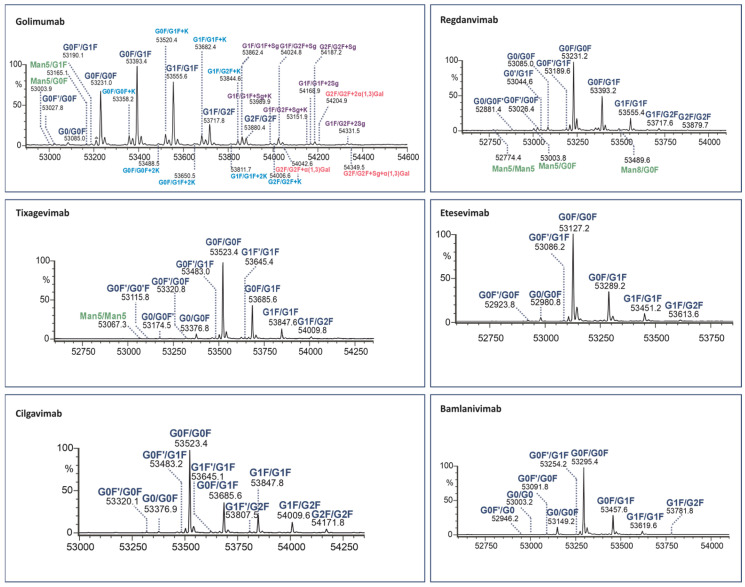
Assignment of glycoforms in the deconvoluted mass spectra of mAbs on Kgp middle-up level by LC-MS.

**Figure 5:**
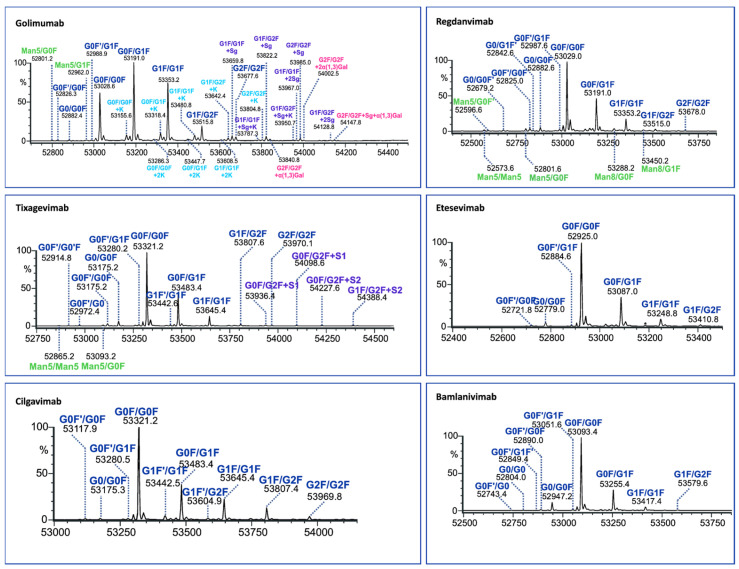
Assignment of glycoforms in the deconvoluted mass spectra of mAbs on IgdE middle-up level by LC-MS.

To elucidate the paired Fc glycans, the LC/MS peak of each Fc fragment was subjected to integration and deconvolution processes. The results displaying the identified isoforms of the six human mAbs (for two middle-up enzymes) are presented in Figure 4 and Figure 5 with detailed information provided in Tables S4 and S5.

Both enzymes cleave IgG1 at adjacent sites of the hinge region. However, IgdE cleaves one threonine amino acid closer to the C-terminus, resulting in Fc masses that are 202 (101 × 2) Da lower than those resulting from KgP digestion.

The identified Fc variants of the mAbs exhibit a range of allotypic diversity and distinct glycoform patterns (Figure 4 and Figure 5). The Fc variants of golimumab and regdanvimab were found to demonstrate the “DELTK” allotype (exp. MW of G0F/G0F is 53,028.8 Da). Tixagevimab and cilgavimab (exp. MW of G0F/G0F is 53,320.9 Da) are recombinant human IgG1κ monoclonal antibodies that contain substitutions in the Fc regions [UK-Evushield, Loo 2022 ]. The M252Y/S254T/T256E (YTE) modification extends the half-life of monoclonal antibodies [Loo 2022] and the L234F/L235E/P331S (TM) modification decreases binding of the Fc receptor and complement component C1q, reducing antibody effector function and the potential risk of antibody-dependent disease enhancement [Loo 2022]. Etesevimab (exp. MW of G0F/G0F is 52,924.6 Da) has abrogated Fc effector function due to the engineered LALA (L234A, L235A) mutation in the heavy chain. This modification can potentially improve the bioavailability and pharmacokinetics of the drug [EMA/177113/2021].

The Fc fragments of the mAbs provided complex patterns consisting of paired glycoforms, which were well resolved under both middle-up conditions. Identifying a large number of Fc variants is crucial for complex antibody products due to the greater diversity of N-glycans present at the glycosylation site of both heavy chains, alongside the almost evenly distributed C-terminal lysine variants. For instance, the UHMU LC-MS technique was used on golimumab, revealing a total of 28 glycoforms, including 10 Fc glycoforms, of which 8 were also associated with the C-terminal lysine. Furthermore, five Fc glycan species were found to exhibit sialylation with N-glycolylneuraminic acid (NGNA), two of which contained unclipped lysine. In addition, three Fc glycan variants showed terminal gal-α-gal linkages. 

The Fc glycoform profiles are generally equivalent between IgdE and Kgp, with small variations. Thus, when using IgdE, there was a tendency to detect a slightly larger number of variants. These trace glycoforms include species with an abundance of around 1% or less, such as high-mannose (HM) glycan types (golimumab, tixagevimab and regdanvimab), sialylated glycans (tixagevimab) or those lacking the terminal N-acetylglucosamine species (G0F’, bamlanivimab).

As expected, reducing the size of mAbs, particularly from 150 to 50 kDa, enhances the characterization of the Fc glycoforms. This is because the associated mass spectra are less complex while still retaining all the information from both chains. In order to demonstrate the benefits of the UHMU approach, the results of paired Fc glycosylation analysis of three different types of mAbs (adalimumab, trastuzumab and rituximab) were compared with their intact MS profiles.

### 3.3. Comparision with Intact MS

**Figure 6:**
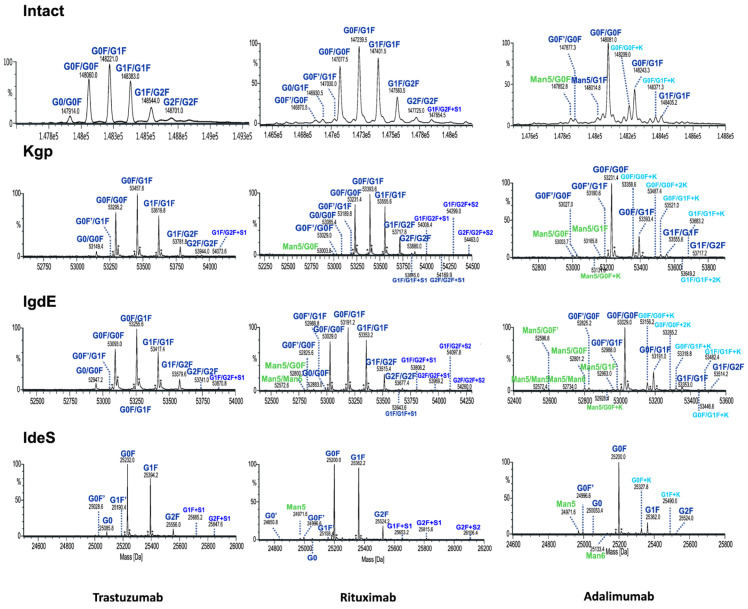
Comparison of deconvoluted mass spectra of intact mAbs or Fc fragments after digestion using IgdE, Kgp or IdeS.

Although both intact and UHMU analyses of three mAbs reveal a similar overall N-glycosylation profile in terms of the main glycoform, notable distinctions arise between the two methodologies. Middle-up LC-MS exhibited narrower MS peaks and greater spectral resolution efficiency of paired glycoforms than intact MS, which enabled the confident identification of a broader spectrum of Fc glycovariants (Figure 6, Table 2). 

The UHMU approach uniquely allows for the identification of low-abundance species not offered by intact MS analysis. In particular, some sialylated, high-mannose (HM) or lysine variants were only detected using middle-up conditions, whereas they were not assigned at the intact MS level (at an accuracy level below 30 ppm). The presence of these forms is also confirmed by middle-up analysis of the Fc/2 spectrum derived using IdeS, e.g., high-mannose Man5 of rituximab, Man5 and Man6 of adalimumab or sialylated species of trastuzumab (Figure S1, Table S2).

The MS data routinely acquired on the Q-TOF instrument consistently yield deconvoluted spectra with a mass error of less than 1 Da from the theoretical masses of proteins with a MW of approximately 50 kDa. The quality of the MS data obtained by UHMU LC-MS allowed mass accuracies of less than 0.5 Da (10 ppm) to be achieved for the majority of the Fc-paired glycoforms with an abundance of >5%.

### 3.4. Quantitative Comparision between Batches

The ability of the UHMD approach to identify mAb glycovariants and reveal differences in the glycoprofile was compared. Three batches of two therapeutic antibodies (trastuzumab and rituximab) with different levels of glycan composition complexity and between-batch glycoprofile variability were evaluated for each proteolysis technique. The relative abundances of each glycoform pair were calculated based on the response (MS peak area) of the individual glycoform relative to the total MS peak areas of all identified glycoforms in the deconvoluted spectra. 

**Figure 7:**
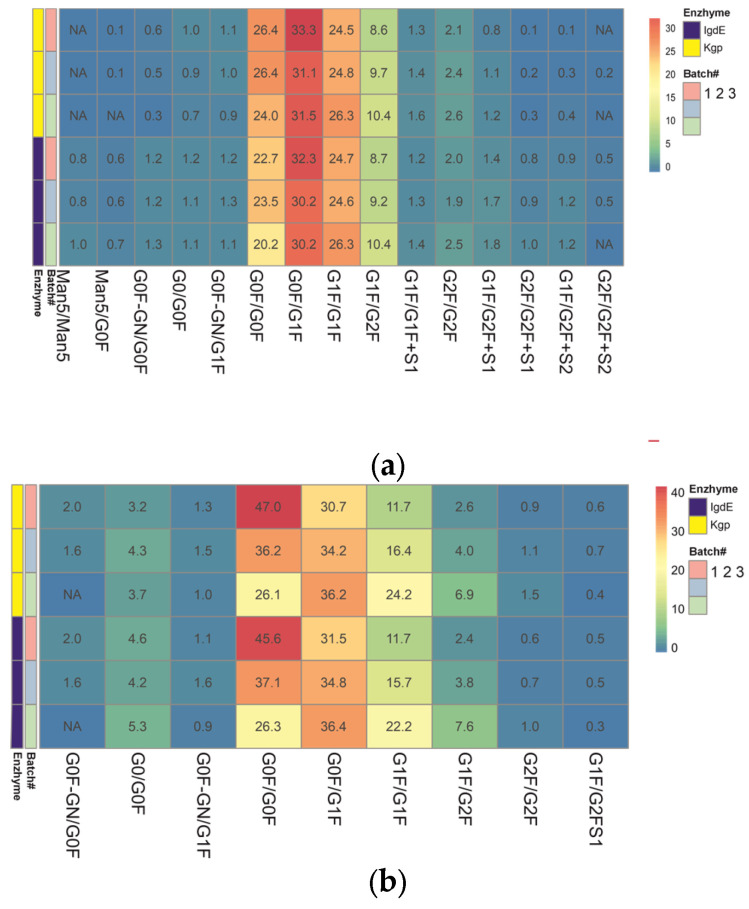
Heatmap representing the color gradient for lowest to highest glycan relative abundance of glycoforms from three batches of each mAb obtained after digestion using IgdE or Kgp: trastuzumab (**a**), rituximab (**b**). ‘NA’ in the cells denotes glycoforms that were not assigned or identified in the analysis.

Heatmaps of pre-organized data constructed from the relative abundances of mAb glycoforms were used to facilitate comparison of the quantitative glycoprofiles (Figure 7). This format allows for the visualization of the distribution of the paired glycovariants in different batches after the protease treatment. Each row of the heatmap corresponds to a different batch of mAb samples analyzed. The columns represent the various glycoforms detected in these samples. The color-coded cells reflect the relative abundance of each glycoform within a batch. The color gradient highlights the distribution range of each glycoform, from low (blue) to high (red) abundance.

The results obtained using both middle-up techniques showed that N-glycosylation varies between trastuzumab batches. The N-glycans showing the most variability are G0F/G0F, G1F/G1F and G1F/G2F (variation between batches of around 30% or more), which means that batches differ in terminal galactosylation. Batch #3 was found to be missing G0F′/G0F glycosylation. The difference in results (delta) between the two procedures did not exceed 10% for the major glycoforms, except for the minor G0/G0F, and trace species G2F/G2F and G1F/G2FS1 (more than 30%).

The relative quantitation of glycoform results for three different batches of rituximab shows good overall comparability between the two UHMU techniques. Minor batch-to-batch variability in sialylated variants was revealed, as G2F/G2F+S2 was not detected in two batches. However, there was a difference in the content of trace glycoforms. Kgp proteolysis failed to detect high-mannose glycoforms with a relative abundance of less than 1% (Man5/Man5 and Man5/G0F), and the levels of agalactosylated glycoforms and sialylated species with a relative abundance of less than 2% (G0/G0F, G0F′/G0F) were underestimated compared to IgdE techniques. The most variable N-glycans identified in samples after IgdE treatment were Man5/Man5, G0F′/G1F and G2F/G2F. The variation between batches was less than 15%.

Both middle-up approaches revealed differences in glycoprofiles between batches of mAbs, although IgdE was more effective in detecting differences in minor glycovariants. The qualitative and quantitative differences in low-expressed glycoforms between the two proteolysis methods may be due to variations in sample preparation techniques, such as buffer choice and reaction time. It is important to note that proteolysis in Kgp cases followed the recommended conditions provided by the manufacturer. However, the use of a weakly reducing buffer and shortened digestion time with the Kgp enzyme may have contributed to incomplete subunit release and the loss of low-intensity glycoforms. Additionally, these mild digestive conditions could potentially result in the partial breakage of the molecule’s disulfide bonds.

## 4. Discussion

Therapeutic mAbs were subjected to selective cleavage above the hinge region using IgG degradation enzymes, specifically IgdE and Kgp, to generate Fc and Fab fragments. Our findings demonstrate the successful cleavage of mAbs and the liberation of distinct subunits suitable for further glycan analysis. IgdE exhibited high specificity and efficiency with minimal unspecific cleavage, while Kgp demonstrated some degree of nonspecific cleavage. This was particularly observable in certain mAbs, such as regdanvimab and bamlanivimab. The CDR3 region is the most variable and plays a crucial role in the diversity and specificity of antigen recognition. The nonspecific Kgp cleavage of mAbs with an extended CDR3 region is an interesting phenomenon that may have some implications for their efficacy and safety as COVID-19 treatments. However, further studies are needed to confirm the occurrence and consequences of these phenomena in vivo and in vitro.

The glycosylation pattern of therapeutic mAbs is a critical quality attribute (CQA) that requires careful monitoring throughout all stages of manufacturing, particularly during process development. Numerous studies have demonstrated the impact of mAb glycosylation patterns on function, stability, efficacy and safety [23,43]. 

It has been shown that changes in the qualitative or quantitative glycan composition can lead to disturbances in antibody immune function [43]. In particular, variants lacking fucose or containing galactose have shown enhanced effector functions [44,45,46,47]. The presence of high-mannose forms or elongation of terminal fragments with galactose can reduce the half-life of antibodies, leading to their rapid elimination from the systemic circulation. These saccharides enhance the interaction of glycoproteins with glycan receptors in the circulation, thereby facilitating their clearance [48,49]. Furthermore, higher levels of mannose-type glycans have been linked to increased antibody-dependent cellular cytotoxicity (ADCC) activity, whereas terminal galactose is known to affect complement-dependent cytotoxicity (CDC) activity [46,47,50,51,52,53]. Finally, the presence of terminal sialic acids resulted in significant immunogenic reactions in humans, accompanied by a decrease in ADCC activity [52,54].

Our findings reveal the potential of LC-HRMS analysis at UHMU level to improve the characterization of mAb N-glycosylation patterns. By selectively cleaving mAbs above the hinge region using enzymes like IgdE or Kgp, this methodology allows for the generation of Fc fragments while conserving the oligosaccharide pairing in the CH2 domain. This enables a more comprehensive assessment of glycoform diversity and abundance, as demonstrated by the identification of low-abundance species not detected by intact MS. For example, using the IgdE technique results in at least nine additional annotated peaks for adalimumab compared to the analysis with intact MS. Four of these peaks related to symmetric HM/HM forms (Man5/Man5 and Man5/Man6) and asymmetric complex (HM/CO) forms (Man5/G0F with lysin variant and Man5/G1F form). The analysis of the adalimumab batches revealed a higher prevalence of asymmetric HM/CO forms compared to symmetric HM/HM forms. Specifically, the relative abundance of Man5/G0F was four times higher than that of Man5/Man5 across all batches. This difference suggests a favored asymmetric pairing due to the predominance of the G0F form compared to Man5 alone (see IdeS). Similar trends were observed among the other mAbs analyzed, all of which contained high-mannose species, except for rituximab. In all three batches, the antibody exhibited slightly elevated levels of symmetric forms.

Previous studies have shown that the IgG glycan type can be the same for both heavy chains (symmetric pairing) or others (asymmetric pairing). Additionally, the coupling process is not completely random, making it impossible to predict the expression of paired forms based on data obtained for individual Fc fragments [55,56,57]. The glycans of the opposing heavy chains interact with each other to maintain the conformation of the IgG Fc domain [55]. Changes in the glycosylation of this region can impact the conformation of the Fc domain and Fc effector functions [32,58,59], as well as the rate of antibody clearance from serum [53]. The study by Liu et al. (2016) found that symmetrically and asymmetrically paired HM forms clear at the same rate, suggesting that the impact on clearance is not directly proportional to the degree of modification [53]. Hence, measuring their relative levels is essential for accurately estimating the effects on clearance, considering their potential coexistence and varying ratios among therapeutic antibodies.

Similarly, this approach was effective in examining the diversity within the C-terminal lysine of the Fc fragment. IgdE proteolysis revealed four additional lysine variants of adalimumab, in contrast to intact MS. This is particularly crucial for highly heterogeneous mAbs that contain combinations of various forms. Our findings indicate that golimumab contains glycans that are not typically found in humans. The glycan composition of mAbs may vary depending on the cell line used for its expression. Chinese hamster ovary (CHO) cells and mouse melanoma-derived cells (SN2/0 or NS0) are commonly used for therapeutic mAb production. Based on current knowledge, the primary IgG glycan structures typically comprise complex, biantennary oligosaccharides that contain N-acetylglucosamine and mannose in the main core structure. These structures are often fucosylated and may have zero, one or two terminal galactose units, along with a minimal amount of sialylation by N-acetylneuraminic acid (NANA). 

Therapeutic mAbs produced in non-human cells may contain non-human glycan epitopes, such as galactosyl-α1–3-galactose (α1–3 Gal) moieties or N-glycolylneuraminic acid (NGNA), a hydroxylated derivative of NANA [60,61,62,63,64]. For example, murine cell lines can express significant amounts of NGNA [62,63]. Moreover, certain CHO cell lines have been found to produce α-Gal epitopes under specific conditions [60]. It has been demonstrated that sialylation with NANA enhances the anti-inflammatory properties of antibodies and reduces their effector functions [52,54]. It is noteworthy that the presence of non-human NGNA sialylation and α1–3 Gal motifs has been linked to compromised product safety due to their potential to induce immunogenic responses, which can lead to adverse patient reactions. 

The relative quantitation of glycoform results across different batches of mAbs revealed good overall comparability between the two UHMU techniques. Minor differences were observed in trace glycoforms, which may be attributed to variations in sample preparation techniques, such as buffer choice and reaction time. 

The analysis revealed significant heterogeneity in galactosylation among the trastuzumab batches. These results suggest that some batches may undergo suboptimal synthesis of complex (galactosylated) N-glycans during the production process.

## 5. Conclusions

In this study, we compared the applicability of the proteases IgdE and Kgp for glycosylation profiling of therapeutic mAbs by LC-MS. The study revealed that IgdE exhibited high specificity, whereas Kgp demonstrated some nonspecific cleavage, particularly in mAbs with extended CDR3 regions.

The upper-hinge middle-up LC-MS analysis provided detailed insights into the paired Fc glycan profiles of the mAbs. The glycoform patterns exhibited considerable diversity among the mAbs, reflecting the complexity of glycosylation in therapeutic antibodies. Notably, the UHMU approach enabled the identification of a broad spectrum of Fc-paired glycovariants with high resolution and sensitivity. This capability is crucial for complex antibody products due to the diversity of N-glycans present at the glycosylation site of both heavy chains.

## Figures and Tables

**Table 1:** Overview of middle-up enzymes utilized in the study.

Specification	IdeS	Gingipain K, Kgp	IgG Degradation Enzyme E, IgdE
Commercial name	IdeS	GingiKhan^®^	FabALACTICA^®^
Biological source	*Streptococcus pyogenes*	*Porphyromonas gingivalis*	*Streptococcus agalactiae*
Expression system	*E. coli*	*E. coli*	*E. coli*
Specificity (IgG1)	..SCDKTHTCPPCAPELLG/GPSV..	..SCDK/THTCPPC..	..SCDKT/HTCPPCP..
Fragments	F(ab′)2(~110 kDa) + Fc/2(~50 kDa)	2Fab(~50 kDa) + Fc(~50 kDa)	2Fab(~50 kDa) + Fc(~50 kDa)
pH	6–8	8	6–8
Buffers	Physiological buffers	2 mM cysteine	Physiological buffers
Reaction time	30 min	1 h	overnight

**Table 2:** Assignment of paired glycan species observed for mAbs after LC-MS analysis at intact and middle-up levels.

mAB	Glycoform	IgdE	Kgp	Intact MS
Trastuzumab	G0/G0F	52,947.3 (9.4)	53,149.6 (11.3)	147,913.5 (25.7)
	G0F′/G1F	53,051.0 (−17.0)	53,254.5 (7.5)	n/a
	G0F/G0F	53,093.0 (0.0)	53,295.4 (3.8)	148,060.4 (31.1)
	G0F/G1F	53,255.5 (7.5)	53,457.6 (5.6)	148,221.0 (20.2)
	G1F/G1F	53,417.6 (7.5)	53,619.8 (7.5)	148,382.6 (16.8)
	G1F/G2F	53,579.7 (5.6)	53,781.9 (5.6)	148,544.6 (16)
	G2F/G2F	53,741.2 (−5.6)	53,944.0 (5.6)	148,700.8 (−24.2)
	G1F/G2FS1	53,870.93 (6.9)	54,073.2 (7.4)	n/a
	Total	8	8	6
Adalimumab	Man5/Man5	52,573.1 (15.2)	n/a	n/a
	Man5/G0F’	52,596.9 (−9.5)	n/a	147,853.0 (9.5)
	Man5/Man6	52,734.7 (4.7)	n/a	n/a
	Man5/G0F	52,801.3 (13.3)	53,003.7 (17)	n/a
	G0F′/G0F	52,825.3 (−5.7)	53,027.2 (−11.3)	147,878.8 (14.9)
	Man5/G0F+K	52,928.5 (−5.7)	53,131.0 (0.0)	n/a
	Man5/G1F	52,962.9 (3.8)	53,165.8 (16.9)	148,015.8 (14.2)
	G0F′/G1F	52,987.8 (0.0)	53,190.5 (9.4)	n/a
	G0F/G0F	53,029.1 (5.7)	53,231.4 (7.5)	148,081.8 (13.5)
	G0F/G0F+K	53,156.2 (−15.0)	53,358.7 (−9.4)	148,210.5 (16.9)
	G0F/G1F	53,191.1 (1.9)	53,393.4 (3.7)	148,244.2 (15.5)
	G0F/G0F+2K	53,285.5 (5.6)	53,487.6 (3.7)	n/a
	G0F/G1F+K	53,318.9 (−3.8)	53,521.1 (−3.7)	148,371.5 (9.4)
	G1F/G1F	53,353.1 (0.0)	53,555.6 (5.6)	148,405.4 (8.8)
	G0F/G1F+2K	53,446.4 (−16.8)	53,649.3 (−3.7)	n/a
	G1F/G1F+K	53,482.3 (18.7)	53,683.5 (0.0)	n/a
	G1F/G2F	53,514.6 (−11.2)	53,717.1 (−7.4)	n/a
	Total	17	14	8
Rituximab	Man5/Man5	52,572.8 (9.5)	n/a	n/a
	Man5/G0F	52,800.7 (1.9)	53,003.8 (19.1)	n/a
	G0F′/G0F	52,825.7 (1.9)	53,028.2 (7.5)	146,869.5 (−13.1)
	G0/G0F	52,882.5 (−3.8)	53,085.7 (15.8)	146,930.6 (14.4)
	G0F′/G1F	52,988.4 (11.3)	53,189.2 (−14.1)	147,030.5 (−20.8)
	G0F/G0F	53,029.0 (3.8)	53,231.5 (8.8)	147,078.3 (25.3)
	G0F/G1F	53,191.2 (3.8)	53,393.7 (8.6)	147,240.0 (22.3)
	G1F/G1F	53,353.4 (5.6)	53,555.7 (8.4)	147,402.0 (21.4)
	G1F/G2F	53,515.4 (3.7)	53,717.8 (6.5)	147,564.0 (20.2)
	G1F/G1F+S1	53,643.7 (−13)	53,845.0 (−11.3)	n/a
	G2F/G2F	53,677.4 (0.0)	53,879.7 (1.7)	147,721.1 (−14.1)
	G1F/G2F+S1	53,806.3 (−3.7)	54,008.8 (18.0)	147,855.7 (23.2)
	G2F/G2F+S1	53,968.96 (6.7)	54,169.9 (−18.5)	148,016.2 (11.9)
	G1F/G2F+S2	54,097.8 (18.5)	54,299.3 (−13.6)	148,146.9 (22.8)
	G2F/G2F+S2	54,260.0 (18.4)	54,463.0 (16.5)	n/a
	Total	15	14	11

## Data Availability

The original contributions presented in the study are included in the article and Supplementary Materials, further inquiries can be directed to the corresponding author.

## Supplementary Materials

The following supporting information can be downloaded at https://www.mdpi.com/article/10.3390/antib13030066/s1, Figure S1: LC/MS profiles (a) and deconvoluted mass spectra of the mAb Fc fragments after digestion using IdeS; Table S1: Overview of biopharmaceuticals used in the study; Table S2: Assignment of species observed for mAbs after LC/MS analysis of IdeS digests; Figure S2: Alignment of sequences for Fc fragments derived from Kgp digestions. Peptides detected experimentally as Kgp putative hydrolysis products are specified by underscored residues; Table S3: Example of putative unspecific Kgp hydrolysis products of cilgavimab; Table S4: Assignment of species observed for mAbs after LC/MS analysis of Kgp digests; Table S5: Assignment of species observed for mAbs after LC/MS analysis of IgdE digests.
