# Supplementary material for: Enhanced N-Glycan Profiling of Therapeutic Monoclonal Antibodies through the Application of Upper-Hinge Middle-Up Level LC-HRMS Analysis
Source: Antibodies (Basel). 2024 Aug 6;13(3):66. doi: 10.3390/antib13030066 (PMC11348383; doi:10.3390/antib13030066)
Supplement: Supplementary file 1 [file antibodies-13-00066-s001.zip › antibodies-3125101-supplementary.pdf]

Supplementary Materials

Enhanced N-Glycan Profiling of Therapeutic Monoclonal Antibodies through the Application of Upper-Hinge Middle-Up Level LC-HRMS Analysis

Natalia Mesonzhnik [1,*], Anton Belushenko [2], Polina Novikova [1], Alexey Kukharenko [3] and Mikhail Afonin [1]

Table S1. Overview of biopharmaceuticals used in the study

mAb	Type	Source	Target	Therapeutic Use	Brand NAME/Manufacturer	Biosimilars
Adalimumab	Human	CHO	TNF-α	Autoimmune conditions	Humira/AbbVie	Amgevita/Amgen, Cyltezo/Boehringer Ingelheim, Hadlima/Samsung Bioepis, Hulio/Fujifilm Kyowa Kirin Biologics, Hyrimoz/Sandoz, Imraldi/Samsung Bioepis, etc.
Golimumab	Human	murine hybridoma cell line	TNF-α	Autoimmune conditions	Simponi/Janssen Biotech	Erelzi/Sandoz, Eticovo/Samsung Bioepis
Regdanvimab	Human	CHO	RBD of SARS-CoV-2	COVID-19	Regkirona/Celltrion	None
Tixagevimab	Human	CHO	RBD of SARS-CoV-2	COVID-19	AZD7442/AstraZeneca	None
Cilgavimab	Human	CHO	RBD of SARS-CoV-2	COVID-19	AZD7442/AstraZeneca	None
Etesevimab	Human	CHO	RBD of SARS-CoV-2	COVID-19	LY-CoV016/Eli Lilly	None
Bamlanivimab	Human	CHO	RBD of SARS-CoV-2	COVID-19	LY-CoV555/Eli Lilly	None
Trastuzumab	Humanized	CHO	HER2	Breast cancer	Herceptin/Genentech	Herzuma/Celltrion, Kanjinti/Amgen, Ogivri/Biocon, Ontruzant/Samsung Bioepis, Trazimera/Pfizer, etc.
Rituximab	Chimeric	CHO	CD20	Non-Hodgkin's lymphoma	Rituxan/Genentech	Blitzima/Celltrion, Rixathon/Sandoz, Riximyo/Mylan, Ruxience/Pfizer, Truxima/Celltrion, etc.

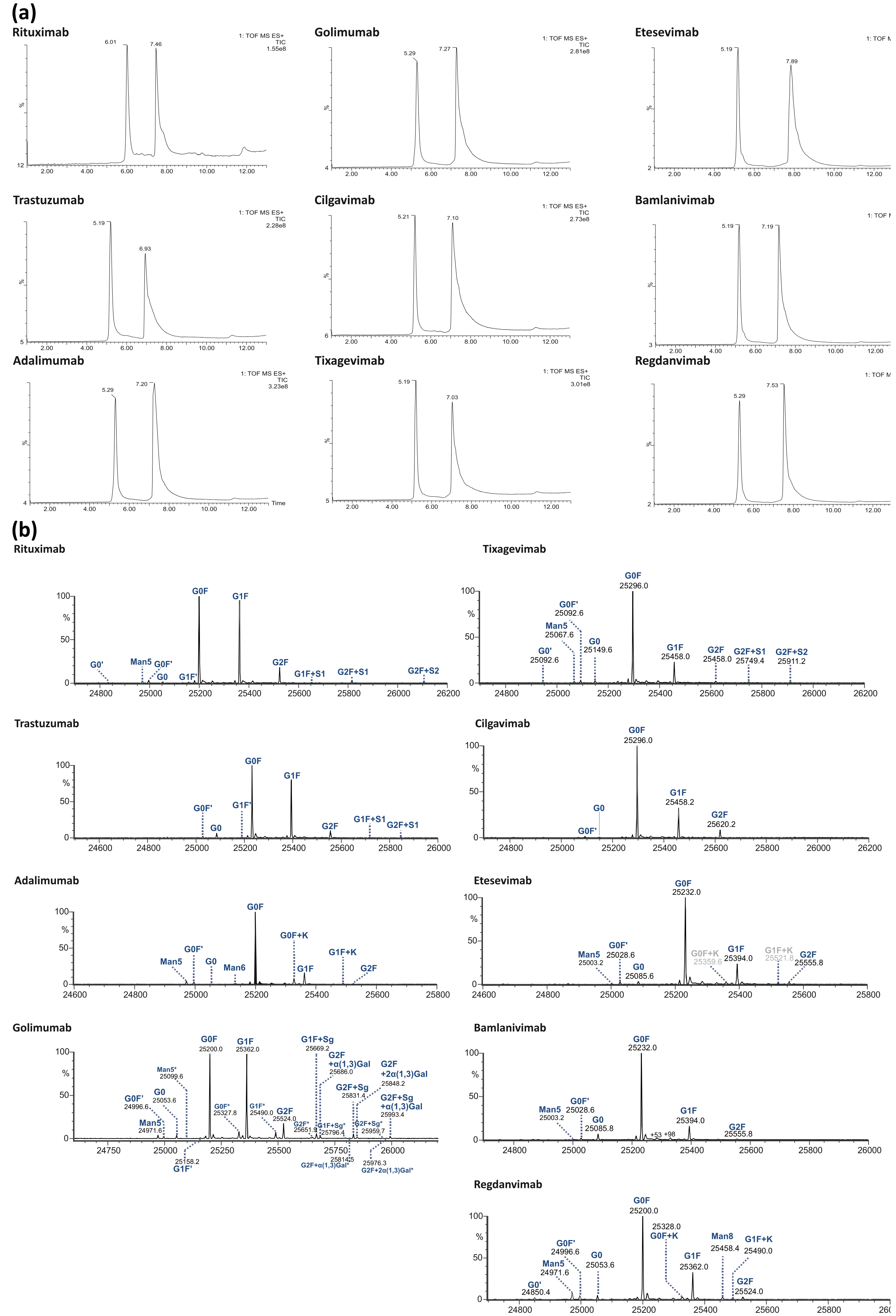

Figure S1. LC/MS profiles (a) and deconvoluted mass spectra (b) of the mAb Fc fragments after digestions using IdeS.

Table S2. Assignment of species observed for mAbs after LC/MS analysis of IdeS digests.

mAb	Glycoform	Expected mass, Da	Observed mass, Da	PPM	% of glycoform
Trastuzumab	G0′	24,882.6	24,882.4	-8.0	0.4
	Man5	25,003.7	25,003.6	-4.0	0.8
	G0F'	25,028.8	25,028.7	-4.0	1.4
	G0	25,085.8	25,085.8	0.0	3.7
	G0F	25,231.9	25,232.1	7.9	46.8
	G1F	25,394.1	25,394.2	3.9	39.7
	G2F	25,556.2	25,556.1	-3.9	6.3
	G2F+SA	25,847.5	25,847.5	0.0	0.6
	G2F+2SA	26,138.7	26,138.2	-19.1	0.3
Adalimumab	Man5	24,971.6	24,971.7	4.0	3.8
	G0F′	24,996.7	24,996.6	-4.0	3.1
	G0	25,053.7	25,053.4	-12.0	0.4
	Man6	25,133.8	25,133.5	-11.9	1.7
	G0F	25,199.9	25,200.1	7.9	65.6
	G0F+K	25,328.1	25,327.9	-7.9	8.4
	G1F	25,362.0	25,362.0	0.0	13.7
	G1F+K	25,490.2	25,489.9	-11.8	1.6
	G2F	25,524.2	25,524.0	-7.8	0.9
Rituximab	G0′	24,850.6	24,850.9	12.1	0.2
	Man5	24,971.6	24,971.7	4.0	1.2
	G0F′	24,996.7	24,996.7	0.0	2
	G0	25,053.7	25,053.6	-4.0	0.9
	G1F′	25,158.8	25,158.5	-11.9	0.8
	G0F	25,199.9	25,200.1	7.9	41.7
	G1F	25,362.0	25,362.2	7.9	41.3
	G2F	25,524.2	25,524.3	3.9	9.3
	G1F+SA	25,653.3	25,653.3	0.0	0.6
	G2F+SA	25,815.4	25,815.7	11.6	1.3
	G2F+2SA	26,106.7	26,106.5	-7.7	0.8
Golimumab	Man5	24,971.6	24,971.6	0.0	1.9
	G0F'	24,996.7	24,996.7	0.0	0.9
	G0	25,053.7	25,053.7	0.0	1.7
	Man5+K	25,099.8	25,099.6	-8.0	0.3
	G1F'	25,158.8	25,158.3	-19.9	0.5
	G0F	25,199.9	25,200.1	7.9	31.3
	G0F+K	25,328.1	25,327.9	-7.9	5.3
	G1F	25,362.0	25,362.1	3.9	33.6
	G1F+K	25,490.2	25,489.9	-11.8	4.7
	G2F	25,524.2	25,524.1	-3.9	7.6
	G2F+K	25,652.3	25,651.9	-15.6	1.1
	G1F+Sg	25,669.3	25,669.2	-3.9	2.8
	G2F+α(1,3)Gal	25,686.3	25,686.0	-11.7	1.6
	G1F+Sg+K	25,796.4	25,796.4	0.0	0.8
	G2F+α(1,3)Gal+K	25,814.4	25,814.5	3.9	0.4
	G2F+Sg	25,831.4	25,831.5	3.9	2.4
	G2F+2α(1,3)Gal	25,848.4	25,848.3	-3.9	1.1
	G2F+Sg+K	25,959.5	25,959.7	7.7	0.4
	G2F+2α(1,3)Gal+K	25,976.5	25,976.3	-7.7	0.3
	G2F+Sg+α(1,3)Gal	25,993.6	25,993.5	-3.8	1.5
Cilgavimab	DEG	23,850.6	23,850.6	-0.4	0
	Man5	25,067.7	25,067.5	-7.6	0.23
	G0F'	25,092.7	25,092.7	0.8	1.27
	G0	25,149.8	25,149.7	-2.8	0.59
	G0F	25,295.9	25,296.2	9.5	58.98
	G1F	25,458.1	25,458.2	4.3	22.4
	G2	25,474.1	25,473.3	-29.8	2.11
	G2F	25,620.2	25,620.2	0.4	6.89
	G2+SA	25,765.3	25,766.0	27.2	0.29
	G2F, glycation	25,782.3	25,781.9	-15.1	0.55
	G2F+SA	25,911.5	25,911.5	2.7	0.8
Etesevimab	Man5	25,003.7	25,003.1	-22.4	0.7
	G0F'	25,028.8	25,028.6	-6.4	2.5
	G0	25,085.8	25,085.6	-7.6	2.9
	G0F	25,231.9	25,232.0	4.0	59.4
	G1F	25,394.1	25,394.0	-3.5	17.5
	G2F	25,556.2	25,555.8	-15.7	2.5
Bamlanivimab	Man5	25,003.7	25,003.2	-20.4	1.0

mAb	Glycoform	Expected mass, Da	Observed mass, Da	PPM	% of glycoform
	G0F'	25,028.8	25,028.6	-4.0	2.4
	G0	25,085.8	25,085.8	0.0	6.7
	G0F	25,231.9	25,232.1	7.1	72.2
	G1F	25,394.1	25,394.0	-2.4	15.7
	G2F	25,556.2	25,555.8	-16.0	2.1
Regdanvimab	G0'	24,850.5	24,850.5	3.2	1.6
	Man5	24,971.6	24,971.7	1.6	5.6
	G0F'	24,996.7	24,996.5	-6.8	3.8
	G0	25,053.7	25,053.6	-5.2	3.5
	G0F	25,199.9	25,200.0	4.4	55.1
	G1F	25,362.0	25,362.0	-0.8	20.4
	G0F+K	25,327.9	25,328.0	4.3	3.8
	Man 8	25,458.1	25,458.4	12.2	2.9
	G1F+K	25,490.0	25,490.2	9.0	1.0
	G2F	25,524.2	25,524.0	-7.4	2.5
Tixagevimab	G0'	24,946.6	24,946.3	-10.0	1.1
	Man5	25,067.7	25,067.7	-0.4	2.2
	G0F'	25,092.7	25,092.6	-4.0	3.4
	G0	25,149.8	25,149.5	-9.1	3.1
	G0F	25,295.9	25,296.1	7.9	63.7
	G1F	25,458.1	25,458.1	1.6	18.7
	G2F	25,620.2	25,620.1	-3.5	3.0
	G1F+S1	25,749.3	25,749.4	2.7	2.2
	G2F+S1	25,911.5	25,911.2	-10.0	1.4
	G2F+S2	26,202.7	26,202.3	-14.9	1.3

.

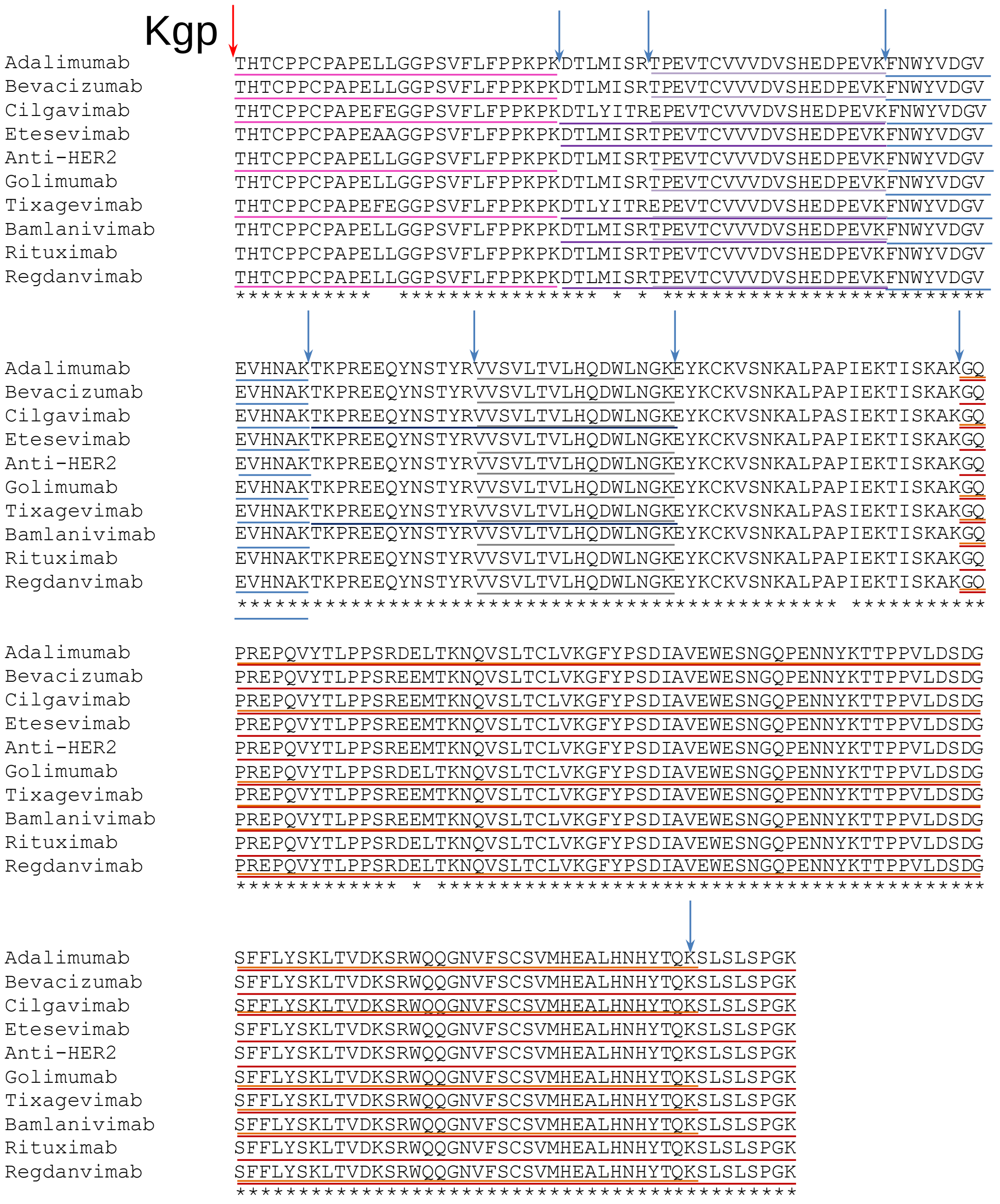

Figure S2. Alignment of sequences for Fc fragments derived from Kgp digestions. Peptides detected experimentally as Kgp putative hydrolysis products are specified by underscored residues. The primary cleavage site is indicated by a red arrow, and putative unspecific cleavage sites by Kgp are indicated by blue arrows.

Table S3. Example of putative unspecific Kgp hydrolysis products of cilgavimab.

RT	m/z	Teor (mono)	z	ppm	Peptide
1.37	559.9391	559.9388	3	0.5	FNWYVDGVEVHNAK
1.84	704.0042	704.0051	3	-1.3	EPEVTCVVVDVSHEDPEVK
3.32	743.8694	743.8721	4	-3.6	DTLYITREPEVTCVVVDVSHEDPEVK
3.77	695.3391	695.3409	4	-2.6	THTCPPCPAPEFEGGPSVFLFPPKPK, 2xCys
4.26	603.3419	603.3403	3	2.6	VVSVLTVLHQDWLNGK
4.86	926.7876	926.7855	6	2.3	THTCPPCPAPEFEGGPSVFLFPPKPK=THTCPPCPAPEFEGGPSVFLFPPKPK
4.9	1259.9451	1259.9455	9	-0.3	GQPREPQVYTLPPSREEMTKNQVSL…WQQGNVFSCSVMHEALHNHYTQK, 2xCys
5.07	1331.2095	1331.2053	9	3.2	GQPREPQVYTLPPSREEMTKNQVSL…WQQGNVFSCSVMHEALHNHYTQKSLSLSPG, 2xCys

Table S4. Assignment of species observed for mAbs after LC/MS analysis of Kgp digests.

mAb	Glycoform	Expected MW	Observed MW	ppm	Da	% of glycosilation
Трастузумаб	G0/G0F	53,149.0	53,149.6	11.3	0.6	3.7
	G0F'/G1F	53,254.1	53,254.5	7.5	0.4	1.0
	G0F/G0F	53,295.2	53,295.4	3.8	0.2	26.1
	G0F/G1F	53,457.3	53,457.6	5.6	0.3	36.2
	G1F/G1F	53,619.4	53,619.8	7.5	0.4	24.2
	G1F/G2F	53,781.6	53,781.9	5.6	0.3	6.9
	G2F/G2F	53,943.7	53,944.0	5.6	0.3	1.5
	G1F/G2FS1	54,072.8	54,073.2	7.4	0.4	0.4
Адалимумаб	Man5/G0F	53,002.8	53,003.7	17.0	0.9	2.1
	G0F'/G0F	53,027.8	53,027.2	-11.3	-0.6	2.3
	Man5/G0F+K	53,131.0	53,131.0	0.0	0.0	0.0
	Man5/G1F	53,164.9	53,165.8	16.9	0.9	1.6
	G0F'/G1F	53,190.0	53,190.5	9.4	0.5	1.6
	G0F/G0F	53,231.0	53,231.4	7.5	0.4	50.4
	G0F/G0F+K	53,359.2	53,358.7	-9.4	-0.5	9.3
	G0F/G1F	53,393.2	53,393.4	3.7	0.2	18.5
	G0F/G0F+2K	53,487.4	53,487.6	3.7	0.2	2.0
	G0F/G1F+K	53,521.3	53,521.1	-3.7	-0.2	3.8
	G1F/G1F	53,555.3	53,555.6	5.6	0.3	4.3
	G0F/G1F+2K	53,649.5	53,649.3	-3.7	-0.2	1.2
	G1F/G1F+K	53,683.5	53,683.5	0.0	0.0	1.4
	G1F/G2F	53,717.5	53,717.1	-7.4	-0.4	1.4
Ритуксимаб	Man5/G0F	53,002.8	53,003.8	19.1	1.0	0.1
	G0F'/G0F	53,027.8	53,028.2	7.5	0.4	0.5
	G0/G0F	53,084.9	53,085.7	15.8	0.8	0.9
	G0F'/G1F	53,190.0	53,189.2	-14.1	-0.8	1.0
	G0F/G0F	53,231.0	53,231.5	8.8	0.5	26.5
	G0F/G1F	53,393.2	53,393.7	8.6	0.5	31.1
	G1F/G1F	53,555.3	53,555.7	8.4	0.4	24.8
	G1F/G2F	53,717.5	53,717.8	6.5	0.3	9.7
	G1F/G1F+S1	53,845.6	53,845.0	-11.3	-0.6	1.4
	G2F/G2F	53,879.6	53,879.7	1.7	0.1	2.4
	G1F/G2F+S1	54,007.8	54,008.8	18.0	1.0	1.1
	G2F/G2F+S1	54,170.9	54,169.9	-18.5	-1.0	0.2
	G1F/G2F+S2	54,300.0	54,299.3	-13.6	-0.7	0.3
	G2F/G2F+S2	54,462.1	54,463.0	16.5	0.9	0.2
Голимумаб	Man5/G0F	53,002.8	53,004.0	22.6	1.2	0.8
	G0F'/G0F	53,027.8	53,027.8	0.0	0.0	0.9
	G0/G0F	53,084.9	53,085.4	9.4	0.5	1.5
	Man5/G1F	53,164.9	53,165.1	3.8	0.2	0.9
	G0F', G1F	53,190.0	53,190.2	3.8	0.2	1.0
	G0F/G0F	53,231.0	53,231.1	1.9	0.1	14.5
	G0F/G0F+K	53,359.2	53,358.2	-18.7	-1.0	3.5
	G0F/G1F	53,393.2	53,393.4	3.7	0.2	19.7
	G0F/G0F+2K	53,487.4	53,488.4	18.7	1.0	1.1
	G0F/G1F+K	53,521.3	53,520.4	-16.8	-0.9	4.2
	G1F/G1F	53,555.3	53,555.6	5.6	0.3	16.8
	G0F/G1F+2K	53,649.5	53,650.5	18.6	1.0	1.3
	G1F/G1F+K	53,683.5	53,682.6	-16.8	-0.9	3.8
	G1F/G2F	53,717.5	53,717.8	5.6	0.3	6.7
	G1F/G1F+2K	53,811.7	53,811.7	0.0	0.0	1.1
	G1F/G2F+K	53,845.6	53,844.6	-18.6	-1.0	2.0
	G1F/G1F+Sg	53,862.6	53,862.4	-3.7	-0.2	3.3
	G2F/G2F	53,879.6	53,880.5	16.7	0.9	3.0
	G1F/G1F+K+Sg	53,990.7	53,989.7	-18.5	-1.0	1.3
	G2F/G2F+K	54,007.8	54,006.8	-18.5	-1.0	1.1
	G1F/G2F+Sg	54,024.7	54,025.0	5.6	0.3	2.8
	G2F/G2F+α(1,3)Gal	54,041.7	54,042.6	16.7	0.9	1.5
	G1F/G2F+Sg+K	54,152.9	54,151.9	-18.5	-1.0	1.0
	G1F/G1F+2Sg	54,169.8	54,168.9	-16.6	-0.9	1.0
	G2F/G2F+Sg	54,186.9	54,187.1	3.7	0.2	1.9
	G2F/G2F+2α(1,3)Gal	54,203.9	54,204.9	18.4	1.0	0.9
	G1F/G2F+2Sg	54,332.0	54,331.5	-9.2	-0.5	1.1
	G2F/G2F+Sg+α(1,3)Gal	54,349.0	54,349.5	9.2	0.5	1.0
Cilgavimab	G0F'/G0F	53,319.9	53,320.1	4.9	0.3	0.9
	G0/G0F	53,376.9	53,376.9	0.2	0.0	1.4

mAb	Glycoform	Expected MW	Observed MW	ppm	Da	% of glycosilation
	G0F'/G1F	53,482.0	53,483.1	19.8	1.1	1.1
	G0F/G0F	53,523.1	53,523.4	7.3	0.4	50.1
	G1F'/G1F	53,644.1	53,645.1	18.3	1.0	1.0
	G0F/G1F	53,685.2	53,685.6	7.3	0.4	19.8
	G1F'/G2F	53,806.3	53,807.3	19.1	1.0	0.7
	G1F/G1F	53,847.3	53,847.8	8.0	0.4	12.9
	G1F/G2F	54,009.5	54,009.7	4.1	0.2	8.4
	G2F/G2F	54,171.6	54,171.7	1.5	0.1	3.8
Etesevimab	G0F'/G0F	52,923.6	52923.8	3.0	0.2	1.1
	G0/G0F	52,980.7	52,980.9	3.8	0.2	3.5
	G0F'/G1F	53,085.8	53,086.2	7.9	0.4	1.0
	G0F/G0F	53,126.8	53,127.2	7.0	0.4	61.1
	G0F/G1F	53,289.0	53,289.2	5.1	0.3	23.7
	G1F/G1F	53,451.1	53,451.3	2.8	0.1	7.5
	G1F/G2F	53,613.3	53,613.7	8.2	0.4	2.0
Bamlanivimab	G0F'/G0	52,945.8	52,946.2	7.2	0.4	0.6
	G0/G0	53,002.9	53,003.2	6.8	0.4	0.6
	G0F'/G0F	53,092.0	53,091.9	-0.8	0.0	1.2
	G0/G0F	53,149.0	53,149.4	6.4	0.3	7.9
	G0F'/G1F	53,254.1	53,254.3	3.0	0.2	1.1
	G0F/G0F	53,295.2	53,295.6	7.9	0.4	62.4
	G0F/G1F	53,457.3	53,457.6	6.0	0.3	20.2
	G1F/G1F	53,619.4	53,619.7	4.8	0.3	4.8
	G1F/G2F	53,781.6	53,781.8	3.5	0.2	1.2
Regdanvimab	Man5/Man5	52,774.5	52,774.4	-2.3	-0.1	1.4
	G0F'/G0	52,881.7	52,881.3	-7.4	-0.4	1.1
	Man5/G0F	53,002.8	53,003.7	18.1	1.0	2.2
	G0F', G0F	53,027.8	53,026.4	-27.3	-1.4	3.1
	G0'/G1F	53,043.8	53,044.6	14.9	0.8	1.4
	G0/G0F	53,084.9	53,085.0	1.5	0.1	3.4
	G0F', G1F	53,190.0	53,189.7	-5.6	-0.3	2.2
	G0F/G0F	53,231.0	53,231.2	3.0	0.2	45.4
	G0F/G1F	53,393.2	53,393.3	2.6	0.1	23.6
	Man8/G0F	53,489.2	53,489.7	9.7	0.5	2.8
	G1F/G1F	53,555.3	53,555.4	1.9	0.1	9.9
	G1F/G2F	53,717.4	53,717.6	3.2	0.2	2.6
	G2F/G2F	53,879.6	53,879.7	1.7	0.1	0.9
Tixagevimab	Man5/Man5	53,066.6	53,067.3	13.8	0.7	0.6
	G0F'/ G0F'	53,116.7	53,115.9	-14.7	-0.8	0.5
	G0F', G0	53,173.7	53,174.5	14.7	0.8	1.5
	G0F', G0F	53,319.9	53,320.7	16.1	0.9	1.2
	G0/G0F	53,376.9	53,376.9	0.0	0.0	4.3
	G0F'/G1F	53,482.0	53,483.0	19.3	1.0	1.3
	G0F/G0F	53,523.1	53,523.5	8.8	0.5	53.4
	G1F', G1F	53,644.1	53,645.4	23.7	1.3	1.1
	G0F/G1F	53,685.2	53,685.7	8.9	0.5	24.1
	G1F/G1F	53,847.3	53,847.7	6.9	0.4	9.0
	G1F/G2F	54,009.5	54,009.6	3.1	0.2	2.9

Table S5. Assignment of species observed for mAbs after LC/MS analysis of IgdE digests.

mAb	Glycoform	Expected MW	Observed MW	ppm	Da	% of glycosilation
Трастузумаб	G0/G0F	52,946.8	52,947.3	9.4	0.5	5.3
	G0F'/G1F	53,051.9	53,051.0	-17.0	-0.9	0.9
	G0F/G0F	53,093.0	53,093.0	0.0	0.0	26.3
	G0F/G1F	53,255.1	53,255.5	7.5	0.4	36.4
	G1F/G1F	53,417.2	53,417.6	7.5	0.4	22.2
	G1F/G2F	53,579.4	53,579.7	5.6	0.3	7.6
	G2F/G2F	53,741.5	53,741.2	-5.6	-0.3	1.0
	G1F/G2FS1	53,870.6	53,870.9	6.9	0.4	0.3
Адалимумаб	Man5/Man5	52,572.3	52,573.1	15.2	0.8	1.1
	Man5/G0F’	52,597.4	52,596.9	-9.5	-0.5	1.1
	Man5/Man6	52,734.5	52,734.7	4.7	0.3	1.0
	Man5/G0F	52,800.6	52,801.3	13.3	0.7	3.3
	G0F'/G0F	52,825.6	52,825.3	-5.7	-0.3	3.4
	Man5/G0F+K	52,928.8	52,928.5	-5.7	-0.3	1.0
	Man5/G1F	52,962.7	52,962.9	3.8	0.2	2.5
	G0F'/G1F	52,987.8	52,987.8	0.0	0.0	1.9
	G0F/G0F	53,028.8	53,029.1	5.7	0.3	46.5
	G0F/G0F+K	53,157.0	53,156.2	-15.0	-0.8	8.5
	G0F/G1F	53,191.0	53,191.1	1.9	0.1	16.1
	G0F/G0F+2K	53,285.2	53,285.5	5.6	0.3	2.2
	G0F/G1F+K	53,319.1	53,318.9	-3.8	-0.2	3.7
	G1F/G1F	53,353.1	53,353.1	0.0	0.0	3.9
	G0F/G1F+2K	53,447.3	53,446.4	-16.8	-0.9	1.4
	G1F/G1F+K	53,481.3	53,482.3	18.7	1.0	1.5
	G1F/G2F	53,515.2	53,514.6	-11.2	-0.6	1.1
Ритуксимаб	Man5/Man5	52,572.3	52,572.8	9.5	-0.5	0.8
	Man5/G0F	52,800.6	52,800.7	1.9	0.1	0.6
	G0F'/G0F	52,825.6	52,825.7	1.9	0.1	1.2
	G0/G0F	52,882.7	52,882.5	-3.8	-0.2	1.1
	G0F'/G1F	52,987.8	52,988.4	11.3	0.6	1.3
	G0F/G0F	53,028.8	53,029.0	3.8	0.2	23.5
	G0F/G1F	53,191.0	53,191.2	3.8	0.2	30.2
	G1F/G1F	53,353.1	53,353.4	5.6	0.3	24.6
	G1F/G2F	53,515.2	53,515.4	3.7	0.2	9.2
	G1F/G1F+S1	53,644.4	53,643.7	-13.0	-0.7	1.3
	G2F/G2F	53,677.4	53,677.4	0.0	0.0	1.9
	G1F/G2F+S1	53,806.5	53,806.3	-3.7	-0.2	1.7
	G2F/G2F+S1	53,968.6	53,969.0	6.7	0.4	0.9
	G1F/G2F+S2	54,096.8	54,097.8	18.5	1.0	1.2
	G2F/G2F+S2	54,259.0	54,260.0	18.4	1.0	0.5
Голимумаб	Man5/G0F	52,800.6	52,801.2	11.4	-0.6	0.6
	G0F’/G0F	52,825.6	52,826.3	13.3	0.7	0.7
	G0/G0F	52,882.7	52,882.3	-7.6	-0.4	1.0
	Man5/G1F	52,962.7	52,962.0	-13.2	-0.7	0.7
	G0F’/G1F	52,987.8	52,988.8	18.9	1.0	1.0
	G0F/G0F	53,028.8	53,028.8	0.0	0.0	15.0
	G0F/G0F+K	53,157.0	53,156.0	-19.8	-1.1	3.5
	G0F/G1F	53,191.0	53,191.2	3.8	0.2	22.0
	G0F/G0F+2K	53,285.2	53,286.2	18.8	1.0	1.4
	G0F/G1F+K	53,319.1	53,318.5	-11.3	-0.6	4.3
	G1F/G1F	53,353.1	53,353.2	1.9	0.1	18.4
	G0F/G1F+2K	53,447.3	53,446.6	-13.1	-0.7	1.4
	G1F/G1F+K	53,481.3	53,480.7	-11.2	-0.6	3.4
	G1F/G2F	53,515.2	53,515.7	9.3	0.5	6.7
	G1F/G1F+2K	53,609.5	53,608.6	-16.8	-0.9	1.0
	G1F/G2F+K	53,643.4	53,642.4	-18.6	-1.0	1.7
	G1F/G1F+Sg	53,660.4	53,659.8	-11.2	-0.6	2.6
	G2F/G2F	53,677.4	53,677.6	3.7	0.2	2.2
	G1F/G1F+K+Sg	53,788.5	53,787.5	-19.5	-1.1	0.8
	G2F/G2F+K	53,805.6	53,804.7	-16.7	-0.9	0.2
	G1F/G2F+Sg	53,822.5	53,822.4	-1.9	-0.1	2.7
	G2F/G2F+α(1,3)Gal	53,839.5	53,840.6	19.5	1.1	1.2
	G1F/G2F+Sg+K	53,950.7	53,949.7	-18.5	-1.0	1.0
	G1F/G1F+2Sg	53,967.6	53,967.0	-11.1	-0.6	1.0
	G2F/G2F+Sg	53,984.6	53,985.0	7.4	0.4	1.9
	G2F/G2F+2α(1,3)Gal	54,001.7	54,002.5	14.8	0.8	0.9
	G1F/G2F+2Sg	54,129.8	54,128.8	-18.5	-1.0	1.3
	G2F/G2F+Sg+α(1,3)Gal	54,146.8	54,147.7	16.6	0.9	1.0

mAb	Glycoform	Expected MW	Observed MW	ppm	Da	% of glycosilation
Cilgavimab	G0F'/G0F	53,117.7	53,117.9	4.0	0.2	1.2
	G0/G0F	53,174.7	53,175.3	11.3	0.6	1.4
	G0F'/G1F	53,279.8	53,280.5	12.8	0.7	1.2
	G0F/G0F	53,320.9	53,321.3	8.6	0.5	49.6
	G1F'/G1F	53,441.9	53,442.5	10.9	0.6	1.3
	G0F/G1F	53,483.0	53,483.4	7.5	0.4	20.4
	G1F'/G2F	53,604.1	53,604.9	14.9	0.8	0.9
	G1F/G1F	53,645.1	53,645.5	6.3	0.3	12.9
	G1F/G2F	53,807.3	53,807.5	5.0	0.3	8.0
	G2F/G2F	53,969.4	53,969.9	9.1	0.5	3.0
Etesevimab	G0F'/G0F	52,721.4	52,721.8	6.8	0.4	1.3
	G0/G0F	52,778.5	52,779.0	9.7	0.5	3.5
	G0F'/G1F	52,883.6	52,884.5	17.0	0.9	1.1
	G0F/G0F	52,924.6	52,925.1	7.9	0.4	59.5
	G0F/G1F	53,086.8	53,087.1	5.8	0.3	23.8
	G1F/G1F	53,248.9	53,249.0	2.4	0.1	7.5
	G1F/G2F	53,411.1	53,410.8	-5.4	-0.3	2.1
	G2F/G2F	53,573.2	53,573.9	12.9	0.7	1.1
Bamlanivimab	G0F'/G0	52,743.6	52,743.5	-3.0	-0.2	0.7
	G0/G0	52,800.7	52,801.1	7.6	0.4	0.6
	G0F'/G1F'	52,848.7	52,849.4	13.2	0.7	0.3
	G0F'/G0F	52,889.8	52,890.0	4.3	0.2	1.5
	G0/G0F	52,946.8	52,947.2	7.6	0.4	7.9
	G0F'/G1F	53,051.9	53,051.5	-6.8	-0.4	1.9
	G0F/G0F	53,093.0	53,093.4	9.2	0.5	61.9
	G0F/G1F	53,255.1	53,255.5	7.9	0.4	19.6
	G1F/G1F	53,417.2	53,417.5	4.7	0.3	4.5
	G1F/G2F	53,579.4	53,579.6	5.0	0.3	1.3
Regdanvimab	Man5/Man5	52,572.3	52,573.6	23.6	-1.3	1.6
	Man5/G0F'	52,597.4	52,596.5	-16.2	-0.8	1.2
	G0F'/G0	52,679.5	52,679.2	-4.9	-0.3	1.2
	G0F'/G0F	52,825.6	52,825.1	-10.6	-0.6	3.0
	Man5/G0F	52,800.6	52,801.6	19.3	1.0	2.5
	G0/G1F'	52,841.6	52,842.6	18.4	1.0	1.0
	G0/G0F	52,882.7	52,882.7	1.1	0.1	3.1
	G0F'/G1F	52,987.8	52,987.7	-0.6	0.0	2.2
	G0F/G0F	53,028.8	53,029.0	4.1	0.2	42.5
	G0F/G1F	53,191.0	53,191.2	3.8	0.2	22.4
	Man8/G0F	53,287.0	53,288.0	18.8	1.0	3.7
	G1F/G1F	53,353.1	53,353.2	2.6	0.1	9.5
	Man8/G1F	53,449.1	53,450.1	18.5	1.0	2.5
	G1F/G2F	53,515.2	53,515.1	-1.9	-0.1	2.4
	G2F/G2F	53,677.4	53,678.0	11.0	0.6	1.2
Tixagevimab	Man5/ Man5	52,864.4	52,865.2	15.3	0.8	0.7
	G0F'/ G0F'	52,914.5	52,914.8	6.0	0.3	0.7
	G0F' /G0	52,971.5	52,972.4	16.8	0.9	0.9
	Man5/G0F	53,092.6	53,093.2	11.1	0.6	1.3
	G0F'/G0F	53,117.7	53,117.6	-0.9	-0.1	2.4
	G0/G0F	53,174.7	53,175.2	9.8	0.5	4.0
	G0F'/G1F	53,279.8	53,280.3	8.4	0.4	1.6
	G0F/G0F	53,320.9	53,321.3	8.8	0.5	49.1
	G1F'/G1F	53,441.9	53,442.6	12.2	0.6	1.3
	G0F/G1F	53,483.0	53,483.5	8.6	0.5	23.3
	G1F/G1F	53,645.1	53,645.5	7.1	0.4	8.0
	G1F/G2F	53,807.3	53,807.6	6.3	0.3	2.1
	G2F/G2F	53,969.4	53,970.1	12.8	0.7	0.7
	G0F/G2F+S1	53,936.4	53,936.5	1.7	0.1	1.3
	G1F/G2F+S1	54,098.5	54,098.6	2.6	0.1	0.9
	G1F/G1F+S2	54,227.6	54,227.6	0.6	0.0	0.8
	G1F/G2F+S2	54,389.7	54,388.6	-19.5	-1.1	1.0
